# Forecasting the Reduction in Urban Air Pollution by Expansion of Market Shares of Eco-Friendly Vehicles: A Focus on Seoul, Korea

**DOI:** 10.3390/ijerph192215314

**Published:** 2022-11-19

**Authors:** Hanghun Jo, Seong-A Kim, Heungsoon Kim

**Affiliations:** Department of Urban Planning and Engineering, Hanyang University, Wangsimri-ro 222, Seongdong-gu, Seoul 04763, Republic of Korea

**Keywords:** eco-friendly vehicles, electric vehicles, hydrogen vehicles, air quality, exhaust gases, vehicular policy, carbon neutrality

## Abstract

Due to global climate change, various countries have agreed upon the use of conventions. In this study, the eco-friendly vehicular policy on carbon neutrality implemented in Seoul, Korea, was examined. To this end, various policy-based scenarios were set, and the changes in automotive exhaust gas emissions were evaluated and compared. The evaluation method combined macroscopic and microscopic emission models as its analysis framework. Micro-traffic data available in Korea were used for analyses, and the results for all autonomous districts were derived to cover the entire area of Seoul. The findings confirmed that the most effective measure is the initial replacement of old, mid-size, or large diesel passenger cars with eco-friendly vehicles (Middle-sized: Scenario 2-1 5.52%, Scenario 2-2 6.86%, Scenario 3-1 80.93%, and Scenario 3-2 83.98%). The replacement of old vehicles exhibited the highest effect in all tested scenarios, while the initial replacement of diesel vehicles was more effective than the replacement of gasoline and liquified petroleum gas vehicles (Diesel: Scenario2-1 6.64%, Scenario 2-2 8.21%, Scenario3-1 86.23%, and Scenario 3-2 90.51%). Among the autonomous districts of Seoul, the Gangnam-gu area exhibited the largest emission-reduced effect among all the tested scenarios (Gangnam-gu: Scenario 2-1 5.80%, Scenario 2-2 6.74%, Scenario 3-1 80.44%, and Scenario 3-2 82.62%). Overall, it was demonstrated that the findings of this study may have significant policy implications in terms of urban emission changes pertaining to transportation.

## 1. Introduction

Rapid urbanization has caused environmental pollution and resulted in an abrupt increase in the number of vehicles and their emissions [1,2]. Vehicles and vehicular traffic are among the largest sources of urban air pollution and greenhouse gas (GHG) emissions [3], constituting the main emission source of various air pollutants, such as carbon monoxide (CO) and nitrogen oxides (NO) [4].

As climatic change became serious owing to air pollution, large cities around the world agreed on carbon neutrality measures to achieve net-zero GHG net emissions (emissions-absorptions) based on the 2015 Paris Agreement. This effort integrated the policies of individual countries to respond to climatic change [5]. Accordingly, the United Kingdom and France legislated their plans to achieve carbon neutrality by 2050, while Korea officially declared the “2050 carbon neutrality” policy in 2020 [6].

According to the World Health Organization, Seoul maintains an extremely poor particulate matter (PM) concentration level (46 μg/m^3^) compared with major cities overseas. In Seoul, vehicle emissions are known to significantly impact air quality, accounting for 85.2% of the emitted air pollutants [7]. Moreover, the deterioration of air quality due to fine dust affects the health of citizens, and the management of air quality is required according to environmental policies [8]. Thus, Seoul has implemented various alternative policies, such as implementing the Green Transport Zone, limiting the operation of outdated diesel vehicles, and managing vehicular operations according to the gas emission classification (grades 1 to 5) to reduce the air pollutants caused by vehicles. The dissemination of eco-friendly transportation indicates that transportation plays an essential role in implementing sustainable policies [9].The city has also expended efforts to disseminate eco-friendly vehicles with no air pollutant emissions, such as those electrically and hydrogen-powered [10]. Recently (June 2021), Seoul presented a policy to accelerate the dissemination of eco-friendly vehicles as one of the leading green mobility strategies for GHG reduction in the “2050 Seoul Climate Action Plan.”

Several studies related to eco-friendly vehicles reflect the changing urban paradigm. In the transportation sector, studies related to emissions from eco-friendly vehicles have been actively conducted in various countries. However, studies on Seoul have not been actively conducted. According to our research, no studies have calculated and predicted emissions based on the actual road conditions in Seoul. In other words, no studies have been performed that accurately reflect the eco-friendly vehicular policy of Seoul or its policy implications or examined whether the policy is effective at reducing emissions. Furthermore, only a few studies performed verification and prediction analyses on the topic of eco-friendly vehicles based on the use of traffic data and emission models. Therefore, this study sets a scenario that reflects the actual policy goals of Seoul, and the fact that the macro- and micro-emission models were combined is considered to be the policy-related and academic contribution of the study.

This study aims to examine the effectiveness of Seoul’s eco-friendly vehicular policy for air pollution reduction and to verify and predict emissions that reflect actual road conditions using Seoul’s traffic data. The detailed goals set in each step to achieve the research objectives are as follows. First, the air pollutant emission factors of eco-friendly and internal combustion engine vehicles (ICEVs) on roads are identified and their emissions are calculated. The emissions from road-mobile pollution sources are calculated using a bottom-up approach (BUA). In addition, the traffic volume of each road section (link) is estimated based on the travel demand forecasting model, and emissions are calculated by applying the road length and emission factor. Next, based on scenarios of emission changes, the changes in air pollutant emissions are identified, and the optimal scenario is selected. The effects of urban air pollution reduction and change caused by the introduction of eco-friendly vehicles are analyzed to calculate policy-related, social, and economic impacts.

**Figure 1:**
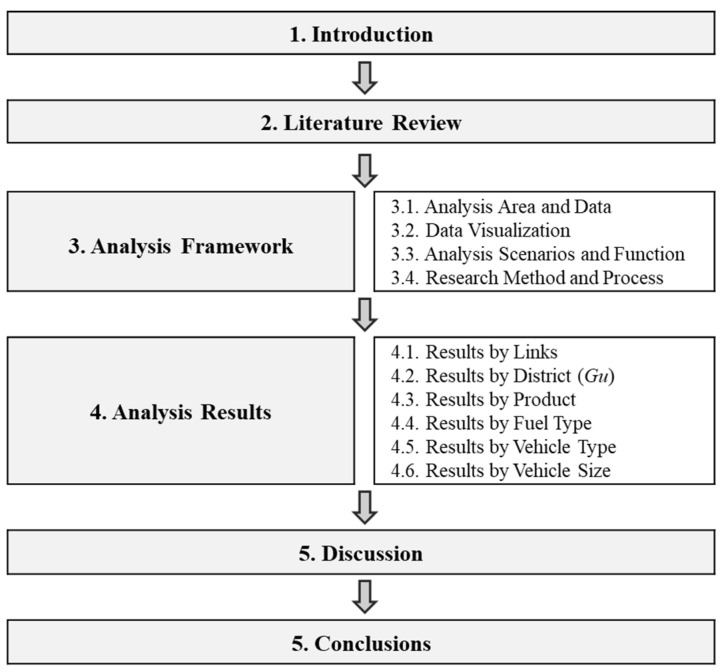
Flowchart showing the process adopted in this study.

Figure 1 shows a flowchart depicting the processes adopted in this study to achieve the objectives listed above. In Section 2, previous studies related to eco-friendly vehicles and automotive exhaust gas emissions are reviewed, and the differences between this study and previous studies are examined. In Section 3, the analyzed data are examined, wherein scenarios are set, and the emission function is selected. An analysis method is then set for the scenarios’ verification, and an analysis process for emission calculation is constructed. In Section 4, the analyzed results for each scenario are presented, and the changes by region, fuel, product, and link in each scenario are examined. Finally, in Section 5, the significance of this study is discussed, and its policy-related and academic implications are proposed.

## 2. Literature Review

Before the analysis, previous studies related to automotive exhaust gas emissions and eco-friendly vehicle research were reviewed. Several previous studies calculated and verified automotive exhaust gas emissions regarding air pollution reduction. Emissions are calculated in various ways depending on the vehicular speed, characteristics, size, production year, and fuel type. Panis et al. [11] and Rodríguez et al. [12] calculated emissions according to vehicular speed, while Osorio and Nanduri [13] and Huang et al. [14] constructed an emissions model that reflected vehicular and regional characteristics. Choi et al. [15] calculated emissions based on the type of vehicle, and Zhang et al. [16] considered traffic signals in calculating emissions. As all these characteristics affect emissions, most previous studies attempted to calculate emissions based on considerations of various characteristics because inaccurate results can be obtained if these characteristics are not considered when calculating emissions. In this regard, Quaassdorff et al. [17] estimated emissions at a microscopic (distance) level based on simulation modeling that considered the actual traffic conditions, average vehicular speed, and traffic density of Madrid’s roundabout.

Emissions models are used for estimating emissions and are classified as microscopic and macroscopic models. Macroscopic models facilitate calculations using computers because data such as the average vehicular speed and traffic flow as a function of link and time period are used in such calculations, but they cannot consider detailed traffic characteristics affecting emissions (e.g., changes in speed, vehicular attributes, and time). Thus, the main analysis targets of macroscopic models are large areas, such as cities. Conversely, microscopic models provide high resolution because detailed data, including the vehicle’s type, age, and the individual vehicular speed in seconds, are used, but they are costly and relatively inefficient. Therefore, the scope of microscopic models is mostly limited to small areas [13,18]. Previous studies have used microscopic [11,19,20,21], macroscopic [22,23,24], and combined models [12,13,25,26,27,28] in various ways depending on their purposes.

Conversely, the studies related to eco-friendly vehicles initially focused on mainly electric vehicles (EVs). Siragusa et al. [29] analyzed the economic and environmental effects associated with the replacement of a delivery van with an EV by comparing ICEVs and EVs. They reported that GHG emissions were reduced by 17% (20 km/day) or 54% (120 km/day) following their replacements. Similarly, Nuez et al. [30] measured and compared CO_2_ emissions from ICEVs (gasoline and diesel) and EVs per km in the Canary Islands in Spain. In their research results, gasoline produced more CO_2_ emissions per km than diesel, and the energy efficiency of EVs was confirmed because they yielded the minimum number of CO_2_ emissions. Boren [31] investigated the energy consumption, cost, and noise of electric buses with respect to their sustainability as part of the public transportation system in Sweden as the number of electric buses increased. Taljegard et al. [32] investigated the possibility of implementing a large-scale road system to increase the EV demand in Sweden and Norway.

The latest policies related to eco-friendly vehicles consider EVs and hydrogen vehicles as eco-friendly vehicles. The Korean Government has expanded the concept of eco-friendly vehicles since 2020 to include hydrogen vehicles as part of the Green New Deal Policy. Following this governmental policy flow, Seoul also added a new project to increase the dissemination of hydrogen vehicles in its 2020 master plan for ”Seoul, the leading city of hydrogen cars.” The latest trend associated with the addition of hydrogen vehicles in the eco-friendly vehicular category has led to increased academic research on hydrogen vehicles. Kim et al. [33] examined the effects of EVs and fuel-cell electric vehicles on the market share of eco-friendly vehicles and estimated the GHG emissions generated when the two types of vehicles consume fuel. Shin et al. [34]. also included hydrogen fuel cell vehicles as an alternative to eco-friendly vehicles and analyzed the preferences of domestic eco-friendly vehicle consumers according to the studied scenario. A few studies have reported a reduction in exhaust gas emissions using eco-friendly fuel in diesel engines. The optimal efficiency for reducing exhaust gas emissions was achieved using biodiesel, a non-polluting fuel used in diesel vehicles [35]. An artificial neural network was used to accurately predict the performance and emissions of engines using biodiesel [36]. In addition, studies in Korea have been mostly focused on the urban dissemination environment for eco-friendly vehicles and the effect of eco-friendly vehicle dissemination on the environment [15,37,38,39,40].

Based on the results of the literature review, this study analyzed the latest eco-friendly vehicle policy of Seoul that considers both hydrogen and electric vehicles as the basis of research. An attempt was also made to apply an approach that supplements the shortcomings of macroscopic and microscopic models to reduce vehicular emissions based on the use of eco-friendly vehicles. In this study, an analysis was conducted by combining the average vehicular speed and traffic volume data according to the link and time period mainly used in macroscopic models, detailed vehicle attributes (e.g., fuel, grade, and type) mainly used in microscopic models, and the link data that include road information in Seoul. Although Seoul was selected as the study target, the results for each of the 25 autonomous districts in Seoul and results according to links were derived.

## 3. Analysis Framework

### 3.1. Analyzed Area and Data

**Figure 2:**
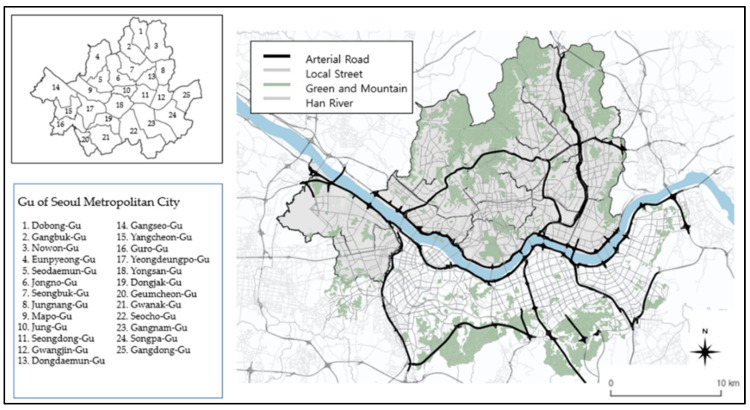
Seoul’s autonomous districts and transportation networks.

The target of this study is Seoul, which is the capital of Korea and the center of the country’s transportation activities. The total area of Seoul is 605.2 km^2^, but the area of land available for urban development is 371.54 km^2^ because the Han River flows through the city from east to west, and high mountains surround the city in the northern and southern areas. Owing to these topographical characteristics, many arterial roads exist in Seoul that efficiently connect the inner and outer parts of the city. They further connect these regions to regions beyond the river and mountains. Seoul comprises 25 autonomous districts and has an internal road network composed of arterial roads and local streets that facilitate transportation within the districts (Figure 2). Seoul is referred to as a vehicle-oriented city owing to its high density of automobile roads even though it is relatively small compared with large cities overseas [41].

Moreover, Seoul has the largest number of registered vehicles in the country, and its traffic volume is concentrated. Thus, various traffic problems and subsequent environmental issues are important policy tasks. According to the “2020 Seoul Air Quality Report,” Seoul exhibited the largest concentration of NOx—which is influenced by road-mobile pollution sources (e.g., vehicles)—among the large cities in Korea, thus indicating that the level of pollutant emissions from vehicles is serious. For these reasons, Seoul was selected as the target of this study.

The data used in this study were mainly transportation network, traffic volume, travel speed, and vehicular attribute data. First, for the transportation network, Seoul’s traffic volume, travel speed data, and the data of View T 2.0 operated by the Transportation Big Data Research Department of the Korea Transport Institute were used. View T 2.0 provides vehicular travel speed, traffic volume, traffic congestion index, and human traffic analysis data for national roads by combining navigation route and public data. Thus, it enables a detailed analysis by date, traveler characteristics, and area. For the vehicular attribute data, the total registered motor vehicles data provided by the Ministry of Land, Infrastructure, and Transport (MOLIT) were used. The data categorized the vehicles registered in Seoul based on the vehicles’ type, size, fuel type, and production year. ICEVs are categorized into gasoline, diesel, liquified petroleum gas (LPG), and hybrid vehicles.

In this study, the aforementioned data were used to accurately calculate emissions and obtain results classified for different urban and vehicular characteristics. These data were also combined; the attribute data were also combined based on links to obtain emission results at high-spatiotemporal resolutions.

### 3.2. Data Visualization

**Figure 3:**
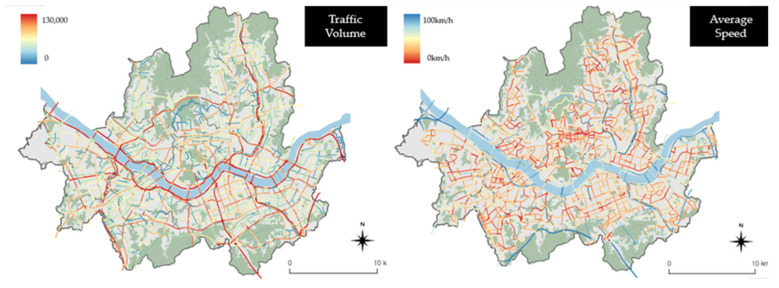
Seoul’s traffic volume (**left**) and average speed (**right**) by the link units.

Before the analysis, the collected data were visualized. First, the traffic volume and vehicular travel speed data provided by View T 2.0 were visualized. These data are highly detailed transportation network data that correspond to the navigation digital map level 5 network, and information on Seoul is provided by using 8133 link units. Based on the traffic volume and vehicular travel speed data classified based on the link units, Figure 3 shows the annual average daily traffic (AADT) (left) and average vehicular speed (right) in Seoul. The AADT confirmed that the traffic volume is significant on the major arterial roads and main downtown areas in Seoul. The average vehicular speed in Seoul was found to be similar throughout the city, and urban highways had certain locations with high average speeds in each section (Figure 3).

**Figure 4:**
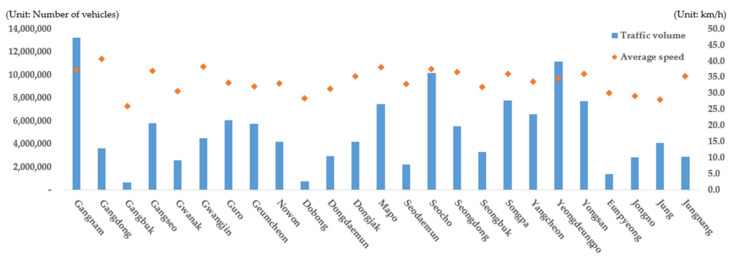
Seoul’s traffic volume and average speed by autonomous districts (*Gu*).

**Figure 5:**
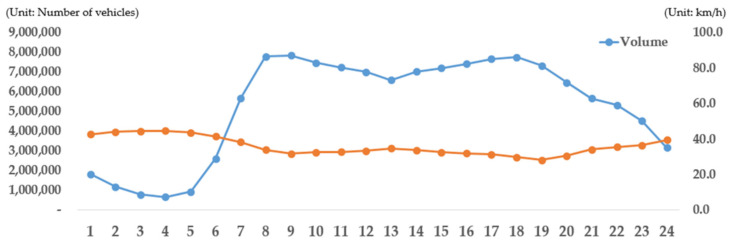
Seoul’s traffic volume and average speed classified by hour.

When the traffic volume and average travel speed were compared among the 25 autonomous districts in Seoul (Figure 4), Gangnam-gu, which is the main downtown area where traffic is concentrated, yielded the largest traffic volume, followed by Yeongdeungpo-gu and Seocho-gu. Gangbuk-gu yielded the smallest traffic volumes, followed by Dobong-gu and Eunpyeong-gu, as these districts are close to mountainous terrain. There was no significant difference in the average travel speed among the districts, but Gangbuk-gu yielded a significantly lower speed (25.9 km/h) compared with other areas. This appears to be because there is a great deal of mountainous terrain and no highways in Gangbuk-gu. When the traffic volume and average travel speed for each time period in Seoul were examined (Figure 5), the rush hours with the largest traffic volume were found to be 08:00 to 09:00 and 18:00 to 19:00, confirming that the traffic volume is the largest during the typical commuting hours in Korea. As the traffic volume increased in the time periods, the average travel speed decreased to some extent even though it did not decrease noticeably.

### 3.3. Analysis Scenarios and Function

For the analysis, three scenarios were set corresponding to the introduction of eco-friendly vehicles in Seoul. The vehicular emissions reduction effects in Seoul were compared and examined in different scenarios. Since the scenarios are based on the actual policy, it is difficult to reflect the constantly changing policy goals in the scenarios. Therefore, in this study, scenarios were set by reflecting the latest policy that researchers can consider.

First, the no-action alternative was set as scenario #1. For scenarios #2 and #3, the 2022 target dissemination goal (80,000 EVs and 4,354 hydrogen vehicles) and the 2030 target dissemination rate (50% of all vehicles with EVs and hydrogen vehicles) presented by Seoul in 2020 were met.

**Scenario #1.**  *No-action alternative.*
**Scenario #2.**  *Seoul disseminates a policy for eco-friendly vehicles (80,000 EVs and 4353 hydrogen vehicles) that accounts for 2.668% of all vehicles by 2022 (Seoul, 2020).*
**Scenario #3.**  *Seoul disseminates policy for eco-friendly vehicles (EVs and hydrogen vehicles) that accounts for 50% of all vehicles by 2030 (Seoul, 2021).*

Equation (1) expresses the emission calculation formula for analyzing the set scenarios. In this study, the emission function developed by Jo and Kim [42] was used. The formula was based on the vehicular exhaust–gas calculation method used in the Clean Air Policy Support System (CAPSS) of the National Institute of Environmental Research of Korea (NIER). Vehicular exhaust–gas is calculated based on the emission function, which is measured differently for each vehicle type and country [42,43,44,45,46]. As the calculation coefficient used in NIER is the national standard model of Korea, it was judged that Jo and Kim’s equation was the most suitable function for this study, as it examines the effect of Seoul’s eco-friendly policy with respect to vehicular emissions. Furthermore, Jo and Kim’s equation [42] can also calculate more accurate vehicular emissions because it combines the traffic volume and vehicular travel speed by link in addition to the calculation coefficient of CAPSS. Therefore, this equation was used in this study to calculate various and specific emissions by vehicle type, production year, fuel type, and product [42,47,48].

(1) $$Total\;Emissions_{i,j,k,l}=\underset{i}{\sum }\underset{j}{\sum }\underset{k}{\sum }\underset{l}{\sum }VKT_{i,j,k,l}\times EF_{i,j,k,l}$$

where *VKT* denotes the vehicle kilometers traveled, *EF* is the emission function, *i* denotes the type of vehicle, *j* denotes the fuel, *k* is the model year, and *l* is the emission product.

### 3.4. Research Method and Process

Regarding the specific analysis method, in this study, road-mobile pollution sources and emissions were calculated using the BUA method. The emission analysis was divided into TDA (e.g., CAPSS and a computer program used to calculate emissions from road transport) and BUA (e.g., transportation demand model and MOVES) depending on the approach. The TDA method is useful in calculating total emissions at the national level by using the total travel distance and average travel speed data of road-mobile pollution sources, but it has limitations in considering travel-behavioral changes owing to the implementation of related policies and analyses of local/road unit emissions. In contrast, the BUA method is used for the evaluation of transportation facility development projects or the preliminary feasibility analysis of transportation infrastructure projects in Korea because it can calculate emissions at a high resolution for regional road-mobile pollution sources. Therefore, the BUA method, especially the macroscopic BUA method, was employed in this study. The macroscopic BUA method can identify emissions for each space by calculating emissions as an emission factor by considering the traffic volume and travel speed of each road section. An attempt was made to supplement the existing shortcomings of the macroscopic BUA method, which applies the average emission factor for some vehicle types without considering the emission factor based on region, vehicle and fuel types, and production year in detail, according to the View T 3.0 data provided by the Transportation Big Data Research Department of the Korea Transport Institute and the vehicular attribute data of the Total Registered Motor Vehicles statistics of MOLIT.

**Figure 6:**
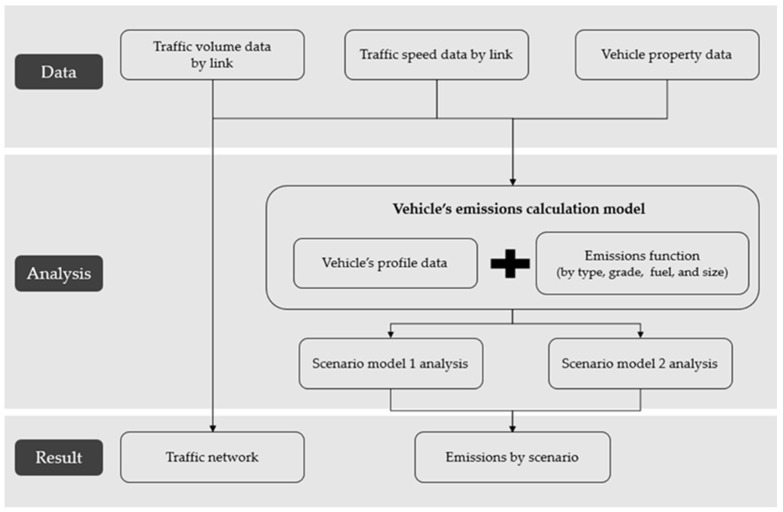
Emissions calculation process.

This study aimed to calculate and analyze air pollutant emissions in each scenario based on the emission factor and big data on transportation, and the analysis was conducted in the following structure (Figure 6). Road network and transportation data were combined by using QGIS (version 3.20) and Excel (version 2016), and emissions were calculated using R (version 4.1.1, manufacturer, Vienna, Austria). First, the link identities of road network data and those of the traffic volume and travel speed were matched and joined (QGIS). The most basic data used to obtain traffic emissions were road network, traffic volume, and travel speed data. In this study, emissions were calculated based on the road network link unit rather than the regional unit to obtain detailed and precise analysis results. Therefore, a road network was constructed by combining road network data with traffic volume and travel speed data. The total road network comprised 8800 roads, the combined traffic volume was the AADT, and the daily average travel speed was inputted. The proportion of vehicles was applied using the property data of the autonomous district to which each link belongs. The proportion of vehicle types was calculated for each of the 25 autonomous districts (Gu) of Seoul, and the proportion of the size to the calculated proportion was calculated. The proportion of fuel was calculated from the previously calculated proportion of the size and type of each vehicle. The proportion according to the vehicle characteristics was finally calculated using a stacked calculation method that applies the proportion according to the production year. The traffic volume for each attribute was estimated by calculating the proportions of each vehicle type, size, fuel type, and production year in the constructed road network, along with the vehicular attribute data, and by applying them to the corresponding traffic volume. Emissions classified based on the type of vehicle, fuel type, product, and link were calculated to identify roads or areas that exhibit the highest air pollutant concentration in each scenario after identifying the vehicle and fuel types that emit the largest amounts of air pollutants. Finally, these results were analyzed to verify the effects of Seoul’s eco-friendly vehicular policy.

## 4. Analysis Results

Scenario #1 set in this study is the no-action alternative, which refers to the current status with no change. Vehicular emissions in Seoul—which are generated when this alternative is selected—were set as scenario 1. Scenario 2 was analyzed based on Seoul’s disseminated policy on eco-friendly vehicles (80,000 EVs and 4353 hydrogen vehicles), which account for 2.668% of all vehicles by 2022. In other words, the analysis was conducted based on the assumption that 84,238 vehicles, which correspond to 2.668% of the total number of registered vehicles in Seoul (3,157,361 vehicles) as of 2020, are replaced with eco-friendly vehicles, i.e., hydrogen vehicles and EVs. Although Seoul set specific target numbers for EVs and hydrogen vehicles, 84,238 eco-friendly vehicles were applied in this study without distinguishing between EVs and hydrogen vehicles because both vehicle types produce zero emissions. Furthermore, scenario 2 was subdivided into scenario 2-1 (ICEVs that correspond to grades 4 and 5 are replaced first) and scenario 2-2 (ICEVs that correspond to grade 5 are replaced first). In scenarios 2-1 and 2-2, it was assumed that vehicles other than those intended to be replaced were maintained, as in scenario 1. Scenario 2 was subdivided because Seoul’s policy is focused on the replacement of old vehicles with eco-friendly vehicles. Subsequently, scenario 3 was set based on Seoul’s policy to “achieve an eco-friendly vehicle substitution rate equal to 50% by 2030.” Scenario 3 was subdivided into scenario 3-1 (50% of all vehicles are replaced with eco-friendly vehicles without classifying grades) and scenario 3-2 (the oldest vehicles from grade 5 are replaced with eco-friendly vehicles).

### 4.1. Results by Links

**Figure 7:**
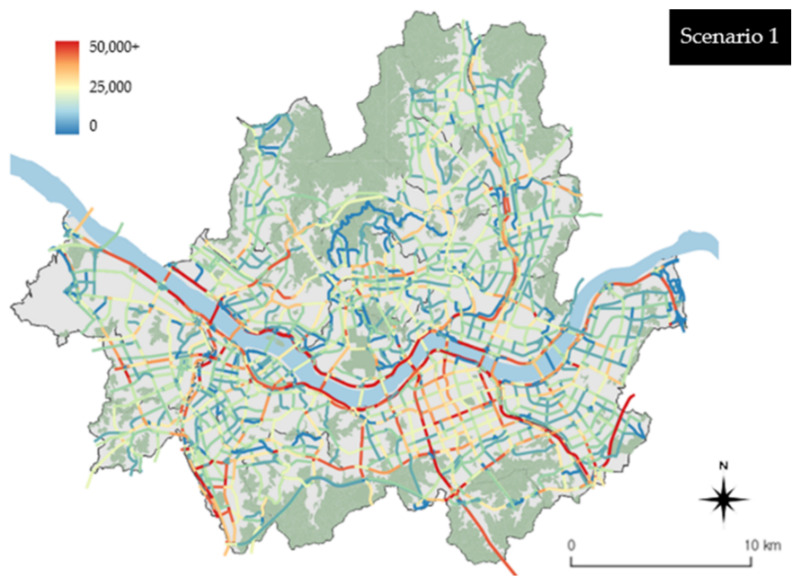
Scenario 1 vehicle emissions by the link units.

When the emission results calculated based on the link units were examined, it was found that high emissions were produced on urban highways and arterial roads in Seoul (refer to Figure 7). The roads that were associated with the highest emissions were urban highways and were located along the Hangang River (e.g., Gangbyeonbuk-ro and Olympic-daero). Both northern and southern sections yielded high emissions. It appears that the roads exhibited high emissions because they have high traffic volumes in Seoul (refer to the traffic volume in Figure 3). Overall, the roads to the south of the Hangang River exhibited higher emissions than those to the north. In particular, the Gangnam-gu area, one of the major downtown areas in Seoul, produced high emissions. This appears to be because the traffic volume is concentrated in the areas south of the Hangang River compared with those north of the river (Figure 7).

**Figure 8:**
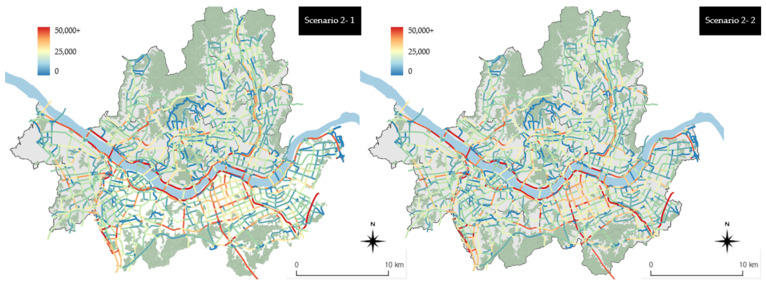
Scenario 2 vehicle emissions by the link units.

Figure 8 shows emissions classified based on the link units in the cases of scenarios 2-1 and 2-2, which are based on the results of scenario 1. A comparison between Figure 7 and Figure 8 shows that emissions decreased throughout the city in the cases of scenarios 2-1 and 2-2 compared with scenario 1. A closer look at scenario 2-1 shows that the overall arterial road emissions did not decrease especially compared with those pertaining to scenario 1, but local street emissions in Jongno-gu, Yeongdeungpo-gu, Seocho-gu, and Gangnam-gu decreased. In the case of scenario 2-2, there are areas where emissions decreased in arterial roads and local streets. Among the arterial roads, Gangbyeonbuk-ro and Olympic-daero, which are located along the Hangang River, exhibited reductions in emissions. Across the city, the emissions associated with high-emission arterial roads were reduced. As in scenario 2-1, emissions were reduced in the cases of local streets in Jongno-gu, Yeongdeungpo-gu, Seocho-gu, and Gangnam-gu. In addition, there were areas where emissions were reduced across the city, including Songpa-gu, Seongdong-gu, and Yangcheon-gu. These results indicate that replacing grade 5 vehicles first (associated with the highest emissions) is more effective for reducing emissions throughout the city than replacing grade 4 and 5 vehicles together with eco-friendly vehicles in the case of scenario 2 (Figure 8).

**Figure 9:**
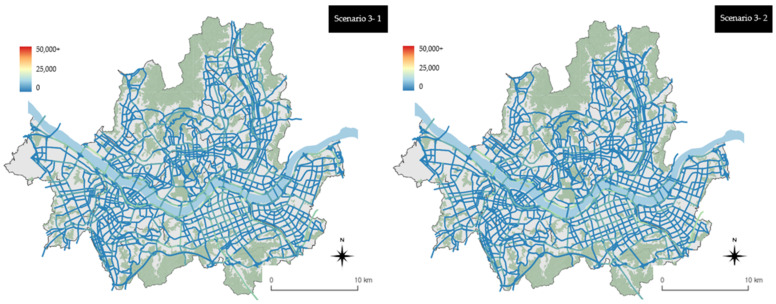
Scenario 3 vehicle emissions by the link units (using the existing range of the legend).

The results for scenarios 3-1 and 3-2 were calculated based on the link units. At the same legend level (as in the previous Figure 7 and Figure 8), the visual differences based on the link units were not significant, as shown in Figure 9. Similarly, scenarios 3-1 and 3-2 also showed no significant differences. These results, however, indicate that there was a very high-emissions reduction effect across the city compared with that in the previous scenario. Both scenarios 3-1 and 3-2 showed emissions ≤5000 g/km^2^, with the exception of the main arterial roads and urban highways of the city. Compared with scenario 3-1 (left side of Figure 9), scenario 3-2 (right side of Figure 9) yielded more links with blue scales indicating low emissions.

**Figure 10:**
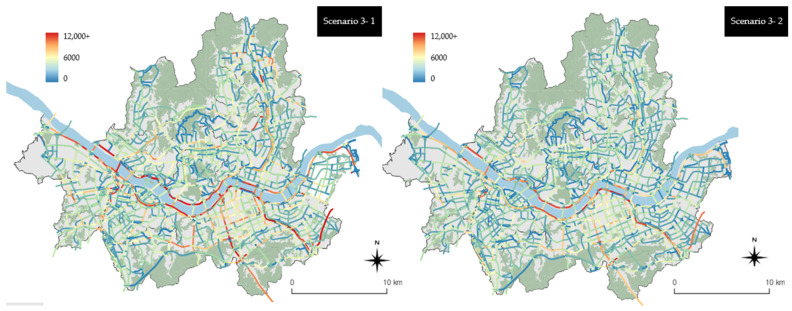
Scenario 3 vehicle emissions by the link units (when changing the range of the legend).

The legend scale was adjusted once more to express scenario 3 at a level that is easier to interpret than that associated with Figure 9 (refer to Figure 10). Figure 10 shows the adjusted legend scale, and the reduction effect can be observed in local streets as well as arterial roads and urban highways in scenario 3-2 compared with scenario 3-1. In particular, it is readily observable that emissions were reduced on the local streets in Gangnam-gu, Nowon-gu, and Yangcheon-gu. It appears that scenario 3-2 had a higher reduction effect because emissions from old vehicles were higher than those from general vehicles of grades 1 to 3 on local streets where the travel speed is usually low. This indicates that local streets yield higher emissions reduction effects than roads with a high travel speed when old vehicles are replaced with eco-friendly vehicles.

### 4.2. Results by District (Gu)

**Figure 11:**
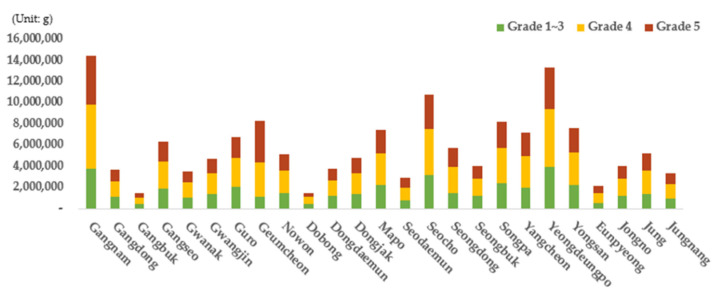
Scenario 1 vehicle emissions by autonomous district (*Gu*) and grade.

**Figure 12:**
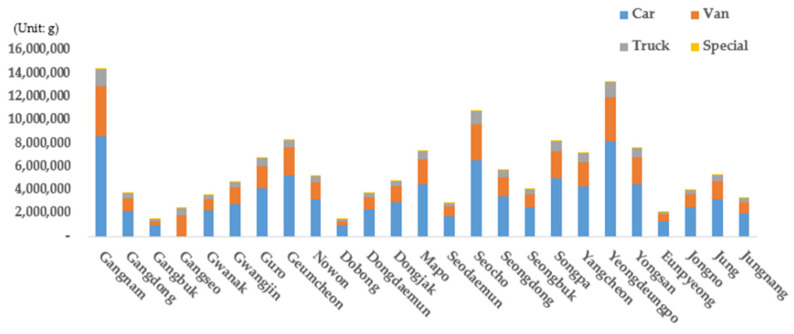
Scenario 1 vehicle emissions by autonomous district (*Gu*) and vehicle type.

**Figure 13:**
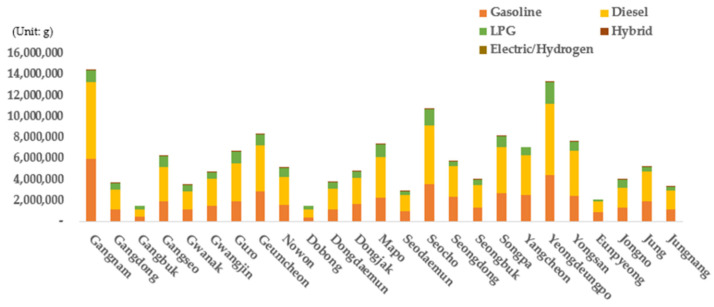
Scenario 1 vehicle emissions by autonomous district (*Gu*) and fuel type.

The data calculated based on the link units were combined for each autonomous district to examine vehicular emissions based on the district in each scenario (Figure 11, Figure 12 and Figure 13). First, when emissions from each autonomous district were examined in scenario 1 (the no-action alternative), Gangnam-gu, Yeongdeungpo-gu, and Seocho-gu exhibited the highest vehicular emissions. These results are similar to the traffic volume results (classified based on the district in Figure 4) because these areas have the largest traffic volumes in Seoul. The traffic volume of Geumcheon-gu, however, was not large compared with other areas, even though its emissions were the highest after those of Gangnam-gu, Yeongdeungpo-gu, and Seocho-gu (refer to Figure 4). Therefore, the emissions for each district were subdivided based on the grade and vehicle and fuel types, and more detailed vehicle emissions were calculated based on considerations of various vehicular characteristics.

First, when the emission results (classified based on the district and grade) were examined (Figure 11), it was found that vehicles of grade 4 yielded the largest proportion of total emissions in Seoul according to the no-action alternative. Vehicles of grades 1 to 3 accounted for 27.8% of the total emissions in Seoul, while grade 4 and 5 vehicles represented 41.0% and 31.2% of the total emissions, respectively. Grades 4 and 5 yielded a significantly larger emission proportion than grades 1 to 3 because old vehicles (corresponding to grades 4 to 5) have a greater impact on emissions even though grades 1 to 3 represent more than half (64.4%) of all registered vehicles in Seoul. For all areas in Seoul, vehicles of grades 4 and 5 accounted for most of the total emissions. In particular, Geumcheon-gu was found to be the district with the highest emissions from vehicles of grades 4 and 5 (86.4%) compared with those of grades 1 to 3. The emission results of scenario 1 by district and grade indicate that the traffic volume of an area as well as the proportions of vehicular grades must be considered to reduce vehicular emissions. In particular, they imply that it is necessary to manage old vehicles of grades 4 and 5.

The emission results were then evaluated based on the district and type of vehicle (Figure 12). Cars yielded the highest emissions among all the types of vehicles. The proportions of emissions from each type of vehicle in Seoul were found to be 60.9% for cars, 28.7% for vans, 9.9% for trucks, and 4.2% for special vehicles. Cars and vans had the largest proportion among all vehicles, followed by trucks and special vehicles owing to the urban characteristics of Seoul. Meanwhile, trucks were associated with the smallest number of registrations, but their emissions were the highest after those associated with cars and vans. This is because trucks produce high levels of emissions, indicating that emissions from trucks cannot be neglected.

When the emissions by district and fuel type in scenario 1 were evaluated (Figure 13), it was found that diesel vehicles yielded the largest emission proportion. The emissions from diesel vehicles exhibited the largest proportion (52.1%) of the total vehicle emissions in Seoul, followed by those from gasoline vehicles (34.4%), LPG vehicles (13.2%), and the emissions from hybrid vehicles (0.16%). It was calculated that the total emissions from EVs was equal to zero because electric motors do not emit any exhaust gases. Among the ICEVs (with the exception of EVs), hybrid vehicles exhibited the smallest emission proportion because they have the smallest number of registrations in Seoul and the use of electric motors at an average travel speed of 30 km/h or less causes zero emissions. In fact, there were numerous links with a speed of 25 to 40 km/h in Seoul when the average travel speed by link was evaluated. The average travel speed was found to be ≤30 km/h in Gangbuk-gu, Dobong-gu, and Chung-gu. Owing to these road characteristics in Seoul, the emissions from hybrid vehicles were found to be zero in many areas.

**Figure 14:**
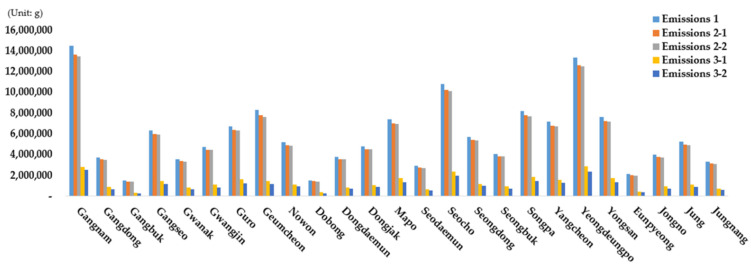
Scenario 2 vehicle emissions and emissions ratio by autonomous district (*Gu*).

The emissions reduction effects associated with the application of scenario 2 were examined based on region, product, fuel, and vehicle type. First, the degree of reduction by district was examined because the proportions of vehicles are different for each district (Figure 14). When scenario 2-1 was compared with scenario 1, Eunpyeong-gu showed the highest emissions reduction effect, followed by Gangnam-gu and Seongdong-gu, while Dongdaemun-gu yielded the lowest effect (left side of Figure 12). In Eunpyeong-gu, which exhibited the largest emissions reduction effect in the case of scenario 2-1, the proportions of diesel and LPG vehicles that correspond to grades 4 and 5 were found to be 0.7% and 2.4% higher, respectively, than the average proportions in Seoul. It appears that Eunpyeong-gu exhibited the highest reduction effect following the replacement of old vehicles of grades 4 and 5 because the proportion of people aged ≥60 years (25.4%) is higher than the average proportion in Seoul (23.5%) [49]. While there was a difference of approximately 0.94%*p* between the district that exhibited the maximum emissions reduction (Eunpyeong-gu 5.97%) and the district that exhibited the minimum reduction (Dobong-gu 5.03%), the average emissions reduction effect of Seoul was found to be 5.22% (compared with that associated with scenario 1) (Table 1).

In the case of scenario 2-2, however, Geumcheon-gu exhibited the highest emissions reduction effect (Figure 14). When the vehicle registration data from 2020 were examined, it was found that a large number of grade-5 LPG vehicles were registered in Geumcheon-gu. Therefore, if grade-5 vehicles are first replaced with eco-friendly vehicles in the case of scenario 2-2, Geumcheon-gu will yield the highest effect compared with other areas. In addition, emissions were reduced by 6.37% on average in Seoul (compared with scenario 1), thus resulting in an approximately 1.08%*p* higher reduction effect compared with scenario 2-1 (Table 1). This is because the emissions from one grade-5 vehicle are greater than those from a corresponding vehicle of a different grade. Therefore, it was confirmed that the replacement of grade-5 vehicles with eco-friendly vehicles has a larger impact on emissions reduction.

Scenarios 3-1 and 1 were compared to examine emissions and the reduction effects (Figure 4 and Table 1). It was found that emissions were reduced by 78.41% in Seoul, and Eunpyeong-gu exhibited the highest reduction effect (80.50%) among the districts. Conversely, Dobong-gu yielded the lowest reduction effect (75.64%) because Eunpyeong-gu and Dobong-gu are associated with low emissions. The largest absolute degree of emissions reduction was observed in Gangnam-gu.

The results of scenario 3-2 are as follows. In the case of scenario 3-2, the reduction effect of Seoul was 82.54%; this was higher compared with that for scenario 3-1. Therefore, replacing old vehicles with eco-friendly vehicles yields a higher exhaust gas reduction effect than reducing the number of all vehicles by 50%. Eunpyeong-gu exhibited the highest reduction effect, followed by Seongdong-gu and Geumcheon-gu. The total emissions reduction was found to be the largest in Gangnam-gu, as in scenario 3-1. It appears that Gangnam-gu exhibited the greatest emissions reduction among all the scenarios because it has high absolute emissions. In addition, Gangbuk-gu, Dobong-gu, and Eunpyeong-gu yielded very low emissions compared with those associated with other districts in the case of scenario 3-2, thus indicating that the emissions from vehicles can be almost eliminated in these districts. Finally, a comparison between the results of scenarios 3-1 and 3-2 revealed that the replacement of old vehicles with eco-friendly vehicles leads to a higher emissions reduction effect than replacing certain proportions of all vehicles with eco-friendly vehicles. In other words, it was confirmed based on this analysis that reducing the number of old vehicles first in a city contributes significantly to emissions reduction.

### 4.3. Results by Product

**Figure 15:**
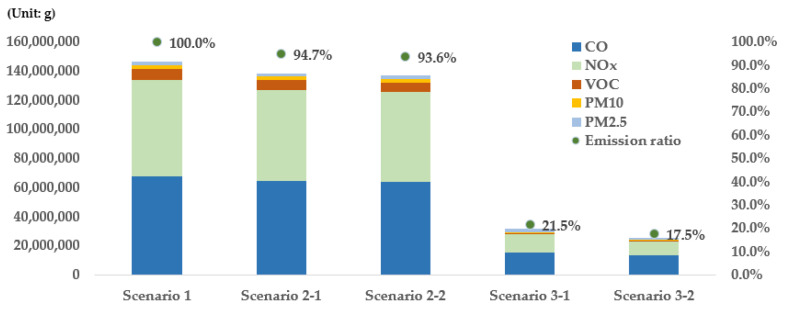
Vehicle emissions and emissions ratio by product.

When the results of scenario 2 were evaluated by product based on scenario 1 (Figure 15 and Table 2), CO (Carbon Oxide) exhibited the largest proportion of total emissions followed by NO_x_ (Nitrogen Oxide), VOC (Volatile Organic Compounds), PM_10_ (Particulate Matter 10), and PM_2.5_ (Particulate Matter 2.5). In the cases of scenarios 2-1 and 2-2, it was found that the proportions of emitted products were not changed significantly compared with those associated with the existing scenario. When the reduction rate was examined for each scenario, NOx exhibited the highest reduction rate (5.57%), while CO yielded the lowest reduction rate (5.02%) in scenario 2-1 (Table 2). CO is emitted from gasoline vehicles, while NOx is emitted from diesel vehicles. In the case of gasoline vehicles, the emission level for each grade is lower than that of diesel vehicles. The emissions from diesel vehicles significantly increase as the vehicles become older. Therefore, the analyzed results indicate that replacing diesel vehicles was more effective at reducing emissions than replacing gasoline vehicles of grades 4 and 5 with eco-friendly vehicles. Unlike scenario 2-1, scenario 2-2 exhibited the highest VOC reduction rate (9.28%), thus indicating that more VOC are emitted from old vehicles compared with other substances. In addition, because PM_10_ and PM_2.5_ are emitted only from diesel vehicles owing to their characteristics, reducing the number of diesel vehicles first is an effective alternative to the reduction of PM emissions.

The results of scenario 3 were examined according to the products (Figure 15 and Table 2). It was found that there were substances whose proportions in emissions changed in the cases of scenarios 3-1 and 3-2 compared with the existing scenario. First, the proportion of CO was 46.4% in the existing scenario case, but it increased to 48.4% in the case of scenario 3-1, and to 53.2% in the case of scenario 3-2. The proportion of NOx, however, decreased from 45.1% in the existing scenario case to 39.6% and 36.5% in the cases of scenarios 3-1 and 3-2, respectively. This indicates that the proportions of substances in emissions may change depending on the method used to replace vehicles with eco-friendly vehicles. When the reduction effect was examined for each scenario, the substance that exhibited the highest reduction effect in scenario 3-1 was found to be NOx (Table 2). In other words, in the case of scenario 3-1, NOx exhibited the highest reduction effect (81.10%), followed by CO (77.54%), VOC (71.23%), PM_10_ (67.30%), and PM_2.5_ (66.88%). In the case of scenario 3-2, NOx also exhibited the highest reduction effect (85.87%), followed by VOC (80.73%), CO (79.98%), PM_10_ (76.82%), and PM_2.5_ (76.05%). These results confirm that replacing old vehicles with eco-friendly vehicles is effective for reducing the substances emitted from diesel vehicles, and it is especially effective for reducing NOx emissions. For all the substances emitted from vehicles (CO, NOx, VOC, PM_10_, and PM_2.5_), the reduction effect of scenario 3-2 was found to be higher than that of scenario 3-1. This result is identical to the result of scenario 2, thus indicating that replacing grade 5 vehicles first with eco-friendly vehicles is the most effective method.

### 4.4. Results by Fuel Type

**Figure 16:**
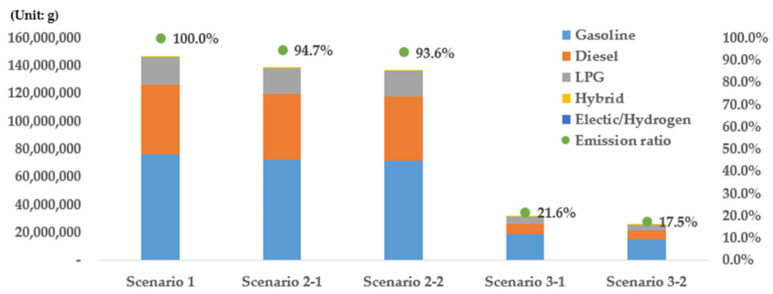
Vehicle emissions and emissions ratios by fuel type.

The emissions for each fuel type and the reduction effects in the cases of scenarios 2 and 3 were examined based on scenario 1 (Figure 16 and Table 3). Among the total emissions, gasoline vehicles exhibited the largest proportion, followed by diesel, LPG, and hybrid vehicles. In the case of the existing scenario 1, the proportion of each type of vehicle in total emissions was found to be 52.1% for gasoline vehicles, 34.5% for diesel vehicles, 13.2% for LPG vehicles, and 0.2% for hybrid vehicles. These proportions did not change in scenarios 2-1 and 2-2. In scenario 2-1, diesel vehicles exhibited the highest emissions reduction effect (6.64%) followed by hybrid vehicles (5.95%), gasoline vehicles (4.79%), and LPG vehicles (3.77%). These results indicate that among the vehicles registered in grades 4 and 5, diesel vehicles exhibited the highest emissions reduction effect. In the case of scenario 2-2, diesel vehicles also yielded the highest emissions reduction effect (8.21%), followed by hybrid (6.31%), gasoline (6.13%), and LPG vehicles (2.56%). It should be noted that scenario 2-2 showed emissions from LPG vehicles and had a lower reduction rate compared with scenario 2-1. This is because the number of grade-5 LPG vehicles is smaller compared with those of grades 1 to 4 as they have shorter lifespans than vehicles that use other fuel types owing to their nature. Therefore, unlike other vehicles, replacing vehicles of grades 4 and 5 was found to be more effective for reducing emissions than replacing only vehicles of grade 5 with respect to LPG vehicles.

When the emissions ratio (classified by fuel type) in the case of scenario 3 was examined, the emissions ratio of gasoline vehicles was found to increase from 52.1% to 58.2% and 64.7% in the cases of scenarios 1, 3-1, and 3-2, respectively (Figure 16 and Table 3). The emissions ratio of LPG vehicles also increased from 13.2% to 19.7% and 16.4% in the cases of scenarios 1, 3-1, and 3-2, respectively. Conversely, the emissions ratio of the diesel vehicles decreased from 34.5% to 22.0% and 18.7% in the cases of scenarios 3-1 and 3-2, respectively. This shows that the replacement of old vehicles with eco-friendly vehicles effectively reduces emissions from diesel vehicles and leads to a higher reduction effect. The reduction effects of scenarios 3-1 and 3-2 were also examined; it was found that both scenarios led to the largest reductions in emissions from diesel vehicles, specifically, equal to 86.23% (scenario 3-1) and 90.51% (scenario 3-2). When these two scenarios were compared, the effect of scenario 3-2 was found to be higher than that of scenario 3-1. The reduction effect of hybrid vehicles was the highest after that of diesel vehicles. In the case of hybrid vehicles, the reduction effect of scenario 3-1 was higher than that of scenario 3-2, unlike vehicles that use other fuel types. This appears to be because the proportions of hybrid vehicles of grades 4 and 5 are small. In addition, the emissions reduction effect of LPG vehicles was found to be lowest in both scenarios 3-1 and 3-2. This is attributed to the fact that LPG vehicles have shorter lifespans than vehicles that use other fuel types; thus, the number of LPG vehicles that correspond to grades 4 and 5 is relatively small.

### 4.5. Results by Vehicle Type

**Figure 17:**
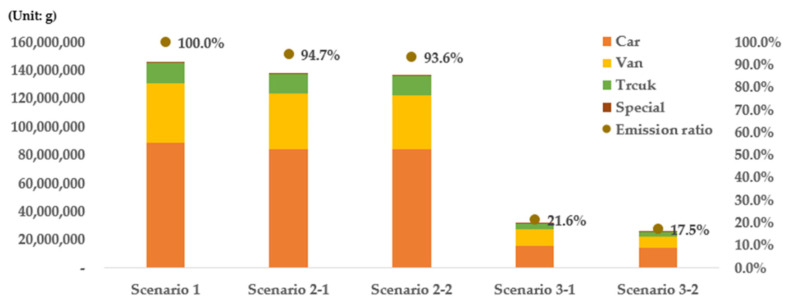
Vehicle emissions and emissions ratios by vehicle type.

In the case of scenario 1, cars exhibited the highest emissions (61.0%), followed by vans (28.7%), trucks (9.9%), and special vehicles (0.4%). In the case of scenarios 2-1 and 2-2, however, the emissions ratio of cars increased to 60.7% and 61.5%, respectively, while emissions decreased for other types of vehicles. This signifies that the emissions reduction values of other types of vehicles were larger than that of cars when the number of old vehicles was reduced. When the reduction rate by scenario was examined, it was found that cars yielded the highest emissions reduction rate (5.66%), followed by vans (4.76%), trucks (4.68%), and special vehicles (3.73%) in the case of scenario 2-1, as shown in Table 4. It appears that cars exhibited the highest reduction effect because the proportions of vehicles of grades 4 and 5 were larger than those of the vehicles of other grades. In the case of scenario 2-2, however, vans yielded the highest emissions reduction rate (8.13%), followed by trucks (6.77%), cars (5.49%), and special vehicles (4.86%). The reduction rate of scenario 2-2 is the most effective at reducing exhaust gas emissions in the city. Regarding special vehicles, such as mixer trucks and excavators, it was found that their emissions were not large because the number of vehicles operating in Seoul is low compared with other types of vehicles, even though the emission factor per vehicle is very high (Figure 17 and Table 4).

When the emissions ratios of all types of vehicles were evaluated in the case of scenario 3, it was found that the emissions ratio of cars decreased from 61.0% in the case of scenario 1 to 50.1% and 57.5% in scenarios 3-1 and 3-2, respectively. The emissions ratios of trucks and special vehicles were found to increase in the case of scenario 3 compared with scenario 1. The emissions ratio of vans increased from 28.7% to 36.2% and 31.1% in scenarios 1, 3-1, and 3-2, respectively. It appears that the total emissions from cars significantly decreased because cars represented 85.9% of all the registered vehicles, and the number of cars replaced with eco-friendly vehicles was greater than those of other types of vehicles in the case of scenario 3. As the total emissions from cars are not high, the emissions ratio of cars was higher than 50%. It was found that cars exhibited the highest emissions reduction effects in both scenarios 3-1 and 3-2 (Table 4). The reduction rate was 82.25% in the case of scenario 3-1, and 83.54% in the case of scenario 3-2. Cars exhibited the highest reduction effect among the types of vehicles because the number of registered cars of grades 4 and 5 is higher compared with those of other vehicles. The types of vehicles other than cars yielded a significant difference in terms of their emissions reduction effects in comparisons between scenarios 3-1 and 3-2. In the case of special vehicles, in particular, the difference between the scenarios was the largest as the reduction effect of scenario 3-2 (78.25%) was 16.4%*p* higher than that of scenario 3-1 (61.85%). Exhaust gas emissions significantly increase as a function of the age of special vehicles. The reduction effect of scenario 3-2 indicates that replacing old vehicles first is more effective for reducing the emissions from vans, trucks, and special vehicles, as demonstrated previously (Figure 17 and Table 4).

### 4.6. Results by Vehicle Size

**Figure 18:**
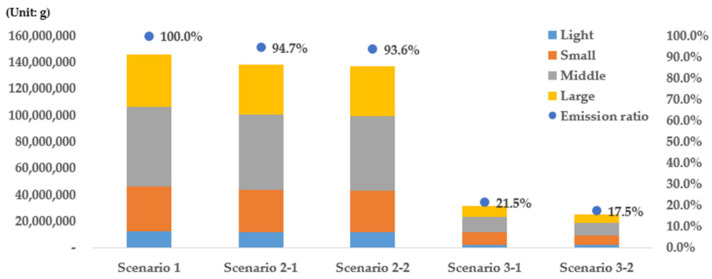
Vehicular emissions and emissions ratios by vehicle size.

When the emissions results concerning the vehicles’ sizes were examined (Figure 18 and Table 5), middle-sized vehicles exhibited the highest emissions ratio (23.1%) in the case of scenario 1 followed by large vehicles (27.2%), small vehicles (23.1%), and compact vehicles (8.5%). This appears to be because the number of middle-sized vehicles is larger than those of vehicles of other sizes. When the emissions reduction effect was examined based on this fact, large vehicles yielded the highest reduction rate (5.63%) in the case of scenario 2-1, followed by middle-sized vehicles (5.52%), compact vehicles (4.94%), and small vehicles (4.62%). In the case of scenario 2-2, however, middle-sized vehicles yielded the highest reduction rate (6.86%) followed by large vehicles (6.2%), small vehicles (6.09%), and compact vehicles (5.34%). The results of scenarios 2-1 and 2-2 can be interpreted as follows. Large vehicles yielded the highest reduction effect in the case of scenario 2-1 and middle-sized vehicles in the case of scenario 2-2 because the proportion of middle-sized vehicles is the highest for grade 5 and that of large vehicles is highest for vehicles with grades 4 and 5 based on the total registered motor vehicles statistics in Seoul. As emissions from large vehicles increase as their age increases, the reduction effect will be high if these vehicles are replaced with eco-friendly vehicles. Based on these results, it was confirmed that replacing larger vehicles first with eco-friendly vehicles is effective for reducing emissions.

When the emissions ratio classified based on vehicular size was evaluated in the case of scenario 3, it was found that the emissions ratios of compact, middle-sized, and large vehicles increased compared with those related to scenario 1. Conversely, the emissions ratio of small vehicles increased compared with those for other vehicles, thus indicating that the emissions reduction effect of other vehicles was higher than those of small vehicles. The same results can also be observed in the emissions reduction ratios for each scenario in Table 5. In addition, when the emissions reduction effect was examined, it was found that the effect of middle-sized vehicles was the highest in the cases of scenarios 3-1 and 3-2. This appears to be because emissions from middle-sized and large vehicles are larger than those from small vehicles, as mentioned above in the case of scenario 2. This indicates that replacing middle-sized and large vehicles with eco-friendly vehicles is effective at reducing emissions regardless of the vehicle’s age. When the emissions reduction effects of scenarios 3-1 and 3-2 were compared for each vehicular size, there was no significant difference in the emissions reduction effect between the scenarios. This result is particularly noticeable for compact vehicles, thus indicating that the influence of old vehicles on emissions is not significant for compact vehicles compared with other vehicular sizes.

## 5. Discussion

In this study, scenarios were set according to Seoul’s policy on carbon neutrality, and the changes in air pollutant emissions from vehicles by scenario were compared and analyzed. The analyzed results of each scenario based on the grade, product, fuel type, and type of vehicle can be summarized as follows (Table 6).

When emissions based on link and district were examined, the Gangnam-gu area exhibited the highest emissions effect in all scenarios. This is because this area has the largest traffic volume in Seoul. Aside from this area, however, the areas were derived slightly differently in each scenario, thus indicating that it is necessary to implement individually customized eco-friendly vehicular policies for each autonomous district rather than an identical policy.

Concerning products, the fuel type to be reduced varies depending on the purpose. If the main purpose is to reduce GHG emissions, it is necessary to replace gasoline vehicles that mainly emit CO with eco-friendly vehicles. To reduce PM, a non-GHG substance, the replacement of diesel vehicles with eco-friendly vehicles was found to be effective. This signifies that the eco-friendly vehicular policy must be set by considering the substance to be reduced because the emitted substance and its amount vary depending on each type of vehicle.

When only the fuel type was considered, it was found that the replacement of diesel vehicles with eco-friendly vehicles had the highest exhaust gas emissions reduction effect. This is because large amounts of PM and NOx emissions—which affect road air pollution—are generated from diesel fuel [28,42,50,51]. Thus, if effective emissions reduction is targeted, it is considered that replacing diesel vehicles first is the most effective policy.

Concerning the types of vehicles, the most effective type for reducing exhaust gas emissions was found to be cars. Kavianipour et al. [52] also concluded that replacing cars to achieve effective exhaust gas reduction constituted the best scenario because cars have the highest market share. In the same context, it appears that the above result was obtained because cars also represent the largest proportion among the registered vehicles in Seoul, in which a large number of old vehicles are registered.

Considering vehicular size, replacing old, middle-sized, or large cars first was found to be most effective at reducing emissions because there are numerous middle-sized or large vehicles. The analyzed results of this study are in agreement with the results of a study by Wang et al. [53], who reported that regulating cars and large vehicles is effective for reducing CO, NOx, and PM emissions; this is because cars are the main cause of CO emissions, while NOx and PM are mainly emitted from large vehicles [42]. In this context, the replacement of cars and large vehicles first is also proposed in this study with respect to implementing policies for eco-friendly vehicles.

Finally, emissions classified by vehicular grade were examined. It was found that replacing old vehicles (grades 4 and 5) first is effective for reducing emissions when ICEVs are replaced with eco-friendly vehicles. This result is in agreement with the results of Kavianipour et al. [52], who reported that replacing old vehicles that produce a high degree of exhaust gas emissions with EVs is the most effective method for CO reductions and that replacing old vehicles aged ≥10 years is the best scenario for overall exhaust gas reduction. In general, the amount of exhaust gas increases as the vehicular age increases [50]. Therefore, replacing old vehicles first is helpful for reducing emissions rather than replacing all grades of vehicles equally when the policy is applied.

Based on the aggregated results, this study derived several policy implications. First, the results derived in this study are significant in that they contributed to identifying the actual effects of the eco-friendly vehicular policy implemented by Seoul. Although previously published studies verified the effect of emissions in different scenarios [52], the scenarios set and evaluated in this study are different in that they reflected the actual policy goals of Seoul rather than the assumptions arbitrarily made by researchers. In particular, the results of this study have significant policy implications because they incorporate newly added policy goals (after the election of Seoul’s new mayor in 2021) in the scenarios.

Second, the most effective method derived for Seoul’s eco-friendly vehicular policy based on the analyzed results of this study involved replacing old, middle-sized, or large diesel cars with eco-friendly vehicles. Old vehicles, particularly old diesel vehicles, exhibited higher exhaust gas emissions than gasoline and LPG vehicles and yielded the best effect in the results of all scenarios in this study. Based on the overall results, it was confirmed that reducing all old vehicles rather than replacing only one vehicle type will help reduce the total amount of emissions and thus lead to improved air quality in the city.

Third, Seoul has implemented various policies in the transportation sector as part of its air pollution policy. These policies mainly involve projects executed to reduce the number of old vehicles, such as the low-pollution measure support project for vehicles of grade 5, old diesel vehicles, and the green transportation area project that limits the operation of grade 5 vehicles. This policy is consistent with the analyzed results of this study that confirmed that the scenarios that first replace old vehicles with eco-friendly vehicles are associated with a higher emissions reduction effect. This observation suggests that Seoul’s policy has been designed effectively, i.e., it is likely to reduce exhaust gas emissions. In other words, it was confirmed that the policy goals of Seoul are effective for reducing vehicular emissions. Seoul also plans to expand the policies currently set only for grade-5 (e.g., the green transportation area project) to grade-4 vehicles in the future [54]. According to this study’s results, grade-4 vehicles represent a considerable proportion of total vehicle emissions in addition to grade-5 vehicles. Therefore, this study empirically confirmed that the expansion of Seoul’s policy to grade-4 vehicles needs to be implemented rapidly.

## 6. Conclusions

Numerous cities have implemented various policies and projects to urgently prepare measures for tackling air pollution and PM reduction. Among these, the introduction of eco-friendly vehicles with no air pollutant emissions constitutes one of the projects that has attracted increased attention. In this context, emissions reduction scenarios were set in this study, and the effects of all the studied scenarios were analyzed to identify the impact associated with the introduction of eco-friendly vehicles on air pollutant emissions. An attempt was made to examine the reduction in vehicular emissions based on the implementation of Seoul’s eco-friendly vehicle policy. To this end, data were obtained by considering various road and transportation characteristics and emissions were estimated based on these data. Furthermore, emissions and the reduction effects were examined for all the studied scenarios based on Seoul’s policy on carbon neutrality.

The academic implications of this study are as follows.

- Air pollutant emissions were calculated from various perspectives based on the type of vehicle, fuel type, product, and link;
- The macroscopic and microscopic emissions models were combined to overcome the limitations in calculating emissions;
- This study provides a basis for future research on identifying the local effects of urban highways and arterial roads because the calculated emissions were classified based on links.

To this end, most of the available microdata in Korea (e.g., average daily speed, traffic volume based on link and time period, and the type of vehicle/fuel type/grade for all 25 autonomous districts) were used for the analysis, and the results based on the district and link were derived rather than obtaining intensive results at the city level. Based on these conditions, the implications at the city level that cannot be identified in the microscopic model and the specific results that cannot be obtained in the macroscopic model were presented. The framework of this study, which derived the results for small areas by integrating various characteristics that can be applied to the urban transportation sector, significantly contributes to research in the transportation sector in that it examines the effects of emissions in a more accurate and specific way.

Based on the conducted analyses, various policy implications were derived in this study. However, the emissions results calculated herein do not represent emissions from actual vehicles that travel in the city, but emissions that were estimated based on the road network, traffic volume, travel speed, and vehicular attribute data. Thus, they may differ from the actual emissions. Moreover, the analysis was conducted by forecasting future policy-based scenarios, and thus uncertainties may exist. In addition, the total amount of exhaust gases emitted in the city may vary depending on the time period (e.g., rush hour, non-rush hour, weekends, and weekdays). However, these time-series data were not considered in this study owing to their nature but are expected to be considered in follow-up research.

## Figures and Tables

**Table 1:** Scenario 2 vehicle emissions and reduction efficiency by autonomous district (*Gu*).

*Gu*	Vehicle Emissions and Reduced Ratios by Scenario								
	Scenario1	Scenario 2-1		Scenario 2-2		Scenario 3-1		Scenario 3-2	
Gangnam	14,455,553	13,617,732	5.80%	13,480,750	6.74%	2,827,919	80.44%	2,512,091	82.62%
Gangdong Gangbuk	3,701,081	3,512,080	5.11%	3,475,378	6.10%	849,397	77.05%	667,739	81.96%
	1,483,945	1,408,629	5.08%	1,385,015	6.67%	327,776	77.91%	266,771	82.02%
Gangseo	6,302,195	5,980,327	5.11%	5,899,339	6.39%	1,436,933	77.20%	1,135,601	81.98%
Gwanak	3,527,361	3,345,190	5.16%	3,315,797	6.00%	810,858	77.01%	630,050	82.14%
Gwangjin	4,707,481	4,464,023	5.17%	4,418,609	6.14%	1,073,241	77.20%	835,220	82.26%
Guro	6,706,231	6,364,783	5.09%	6,296,096	6.12%	1,584,743	76.37%	1,204,111	82.04%
Geumcheon	8,283,556	7,811,454	5.70%	7,635,071	7.83%	1,423,847	82.81%	1,154,607	86.06%
Nowon	5,160,570	4,887,538	5.29%	4,844,800	6.12%	1,122,969	78.24%	909,211	82.38%
Dobong Dongdaemun	1,493,057	1,417,986	5.03%	1,407,085	5.76%	363,777	75.64%	271,496	81.82%
	3,738,677	3,546,149	5.15%	3,519,396	5.87%	837,628	77.60%	678,709	81.85%
Dongjak	4,786,325	4,513,081	5.71%	4,487,437	6.24%	1,043,674	78.19%	845,383	82.34%
Mapo	7,384,565	6,980,532	5.47%	6,913,939	6.37%	1,724,298	76.65%	1,327,111	82.03%
Seodaemun Seocho	2,887,258	2,734,376	5.30%	2,707,024	6.24%	625,480	78.34%	506,945	82.44%
	10,766,976	10,206,963	5.20%	10,108,956	6.11%	2,346,470	78.21%	1,924,070	82.13%
Seongdong	5,713,132	5,393,164	5.60%	5,339,854	6.53%	1,157,012	79.75%	981,077	82.83%
Seongbuk	4,047,718	3,835,963	5.23%	3,799,003	6.14%	903,601	77.68%	717,200	82.28%
Songpa	8,196,381	7,770,408	5.20%	7,693,912	6.13%	1,848,453	77.45%	1,449,980	82.31%
Yangcheon	7,139,073	6,761,376	5.29%	6,692,765	6.25%	1,547,834	78.32%	1,252,321	82.46%
Yeongdeungpo	13,333,721	12,614,216	5.40%	12,499,340	6.26%	2,852,248	78.61%	2,353,816	82.35%
Yongsan	7,609,773	7,211,472	5.23%	7,137,594	6.20%	1,703,698	77.61%	1,354,183	82.20%
Eunpyeong	2,115,653	1,989,365	5.97%	1,977,279	6.54%	412,561	80.50%	359,197	83.02%
Jongno	3,980,872	3,775,959	5.15%	3,718,304	6.60%	912,664	77.07%	704,478	82.30%
Jung	5,249,433	4,969,066	5.34%	4,917,201	6.33%	1,088,778	79.26%	891,957	83.01%
Jungnang	3,309,709	3,135,459	5.26%	3,101,191	6.30%	718,621	78.29%	575,308	82.62%
Seoul	146,080,296	138,347,289	5.29%	136,771,136	6.37%	31,544,479	78.41%	25,508,631	82.54%

**Table 2:** Vehicle emissions and reduction efficiency by product.

Product.	Vehicle Emissions and Reduced Ratio by Scenario								
	Scenario 1	Scenario 2-1		Scenario 2-2		Scenario 3-1		Scenario 3-2	
CO	67,744,364	64,342,624	5.02%	63,689,075	5.99%	15,214,582	77.54%	13,561,458	79.98%
NOx	65,935,001	62,264,345	5.57%	61,645,148	6.51%	12,459,996	81.10%	9,313,589	85.87%
VOC	7,506,129	7,095,590	5.47%	6,809,501	9.28%	2,159,807	71.23%	1,481,156	80.27%
PM_10_	2,588,975	2,455,825	5.14%	2,447,063	5.48%	846,502	67.30%	600,229	76.82%
PM_2.5_	2,305,827	2,188,905	5.07%	2,180,348	5.44%	763,593	66.88%	552,199	76.05%

CO: Carbon Oxide; NOx: Nitrogen Oxide; VOC: Volatile organic compounds; PM: Particle matter.

**Table 3:** Vehicle emissions and reduction efficiency by fuel type.

Fuel	Vehicle Emissions and Reduced Ratios by Scenario								
	Scenario 1	Scenario 2-1		Scenario 2-2		Scenario 3-1		Scenario 3-2	
Gasoline	76,166,402	72,517,851	4.79%	71,499,696	6.13%	18,351,893	75.91%	16,500,533	78.34%
Diesel	50,329,029	46,987,203	6.64%	46,197,006	8.21%	6,927,917	86.23%	4,776,995	90.51%
LPG	19,346,216	18,617,778	3.77%	18,850,851	2.56%	6,229,534	67.80%	4,193,576	78.32%
Hybrid	238,649	224,457	5.95%	223,583	6.31%	35,136	85.28%	37,527	84.28%
Electric/ Hydrogen	-	-	-	-	-	-		-	

Gasoline: a type of petroleum, mainly used as fuel for automobiles; Diesel: a type of petroleum mainly used as a fuel for automobiles—diesel engines have a different fuel injection system than gasoline engines; LPG: liquefied petroleum gas; Hybrid: vehicles that are powered by both electric motors and engines; Electric/Hydrogen: vehicles that use electricity or hydrogen and are eco-friendly vehicles.

**Table 4:** Vehicle emissions and reduction efficiency by vehicle type.

Type	Vehicle Emissions and Reduced Ratios by Scenario								
	Scenario 1	Scenario 2-1		Scenario 2-2		Scenario 3-1		Scenario 3-2	
Car	89,056,745	84,018,643	5.66%	84,164,907	5.49%	15,803,809	82.25%	14,660,506	83.54%
Van	41,896,438	39,903,759	4.76%	38,490,780	8.13%	11,430,209	72.72%	7,934,312	81.06%
Truck	14,503,219	13,824,254	4.68%	13,521,871	6.77%	4,072,438	71.92%	2,778,134	80.84%
Special	623,894	600,634	3.73%	593,577	4.86%	238,023	61.85%	135,679	78.25%

Car: vehicle transporting people; Van: a multi-seater vehicle larger and capable of transporting more people than a car; Truck: vehicle for transporting goods; Special: vehicles for construction or special purposes.

**Table 5:** Vehicular emissions and reduction efficiency by vehicle size.

Size	Vehicle Emissions and Reduced Ratio by Scenario								
	Scenario 1	Scenario 2-1		Scenario 2-2		Scenario 3-1		Scenario 3-2	
Light	12,363,559	11,752,690	4.94%	11,703,117	5.34%	2,552,904	79.35%	2,432,927	80.32%
Small	33,684,441	32,127,024	4.62%	31,633,509	6.09%	9,133,824	72.88%	6,765,629	79.91%
Middle	60,350,986	57,022,029	5.52%	56,212,452	6.86%	11,511,562	80.93%	9,668,721	83.98%
Large	39,681,309	37,445,547	5.63%	37,222,058	6.20%	8,246,189	79.22%	6,641,353	83.26%

Light: vehicle displacement < 1000 cc; Small: 1000 cc ≤ vehicle displacement <1600 cc; Middle: 1600 cc ≤ vehicle displacement < 2000 cc; Large: Vehicle displacement > 2000 cc.

**Table 6:** Summary of results on vehicle emissions and reduction efficiency by scenarios.

Characteristics		Vehicle Emissions and Reduced Ratios				
		Scenario 1	Scenario 2-1	Scenario 2-2	Scenario 3-1	Scenario 3-2
Product	CO	67,744,364	64,342,624	63,689,075	15,214,582	13,561,458
	NOx	65,935,001	62,264,345	61,645,148	12,459,996	9,313,589
	VOC	7,506,129	7,095,590	6,809,501	2,159,807	1,481,156
	PM_10_	2,588,975	2,455,825	2,447,063	846,502	600,229
	PM_2.5_	2,305,827	2,188,905	2,180,348	763,593	552,199
Fuel	Gasoline	76,166,402	72,517,851	71,499,696	18,351,893	16,500,533
	Diesel	50,329,029	46,987,203	46,197,006	6,927,917	4,776,995
	LPG	19,346,216	18,617,778	18,850,851	6,229,534	4,193,576
	Hybrid	238,649	224,457	223,583	35,136	37,527
	Electric/Hydrogen	-	-	-	-	-
Type	Car	89,056,745	84,018,643	84,164,907	15,803,809	14,660,506
	Van	41,896,438	39,903,759	38,490,780	11,430,209	7,934,312
	Truck	14,503,219	13,824,254	13,521,871	4,072,438	2,778,134
	Special	623,894	600,634	593,577	238,023	135,679
Size	Light	12,363,559	11,752,690	11,703,117	2,552,904	2,432,927
	Small	33,684,441	32,127,024	31,633,509	9,133,824	6,765,629
	Middle	60,350,986	57,022,029	56,212,452	11,511,562	9,668,721
	Large	39,681,309	37,445,547	37,222,058	8,246,189	6,641,353

## Data Availability

Not applicable.
